# Chromatin modifications and dynamics during repair of a double-strand chromosome break in budding yeast

**DOI:** 10.1186/1756-8935-6-S1-O16

**Published:** 2013-03-18

**Authors:** James E Haber, Cheng-Sheng Lee, Kihoon Lee, Michael Tsabar, Wade Hicks, Gaelle Legube

**Affiliations:** 1 Rosenstiel Center and Department of Biology, Brandeis University, Waltham MA 02454, USA; 2 Université de Toulouse, UPS, LBCMCP, Toulouse, France

## 

A single double-strand break (DSB) triggers the ATR/ATM (Mec1/Tel1)-dependent DNA damage response in budding yeast. These kinases phosophorylate not only histone H2A (γ-H2AX) but also the C-terminal threonine of histone H2B (γ-H2B), both of which contribute to checkpoint responses and repair of the DSB. Despite both being phosphorylated by the same kinases, γ-H2AX kinetics are much more rapid than that of γ-H2B, but when H2A is mutated to prevent its phosphorylation, γ-H2B kinetics adopt the rapid kinetics seen for γ-H2AX. Both modifications are removed the PP4 phosphatase. γ-H2AX and γ-H2B spread over ≥ 50 kb on either side of the DSB but they are nearly absent in strongly transcribed regions. When transcription is turned off, γ-H2AX rises to a high level within 10 min, catalyzed by either Mec1 or Tel1, even 5 h after the break was induced and when Tel1 is reported no longer to be associated with the DSB. We show that if a DSB is created within 15 kb of one yeast centromere that γ-H2AX and γ-H2B spread to the pericentromeric regions of all other chromosomes, demonstrating that the kinases can act in trans to regions of DNA that are located in close proximity.

When a DSB is created in a locus that can be repaired by gene conversion, 5’ to 3’ resection leads to the loss of well-positioned nucleosomes adjacent to the DSB. When repair is complete, nucleosomes are re-established, but the pattern is distinct from that seen prior to the break or when the repaired cells are allowed to replicate. Chromatin re-establishment is dependent on the histone chaperones CAF-1 and Asf1.

